# Reporting of determinants of health inequities and participant characteristics in randomized controlled trials of juvenile idiopathic arthritis in Canada: a scoping review

**DOI:** 10.1186/s12969-023-00917-5

**Published:** 2023-11-06

**Authors:** Niloofar Gheshlaghi, Megan Thomas, Natasha Trehan, Mark Harrison, Mary A. De Vera

**Affiliations:** 1 Faculty of Pharmaceutical Sciences, University of British Columbia, 2405 Wesbrook Mall, V6T 1Z3 Vancouver, BC Canada; 2 Collaboration for Outcomes Research and Evaluation, Vancouver, BC Canada; 3 Faculty of Science, University of Ottawa, Ottawa, ON Canada; 4 Arthritis Research Canada, Vancouver, BC Canada; 5 Centre for Health Evaluation and Outcome Sciences, Vancouver, BC Canada

**Keywords:** Juvenile idiopathic arthritis, Randomized Controlled Trial, Health inequities, Inflammatory Disease

## Abstract

**Background:**

Juvenile Idiopathic Arthritis (JIA) is the most common form of childhood inflammatory arthritis. The disease burden of JIA is substantial as patients require specialized medical practitioners for diagnosis and chronic treatments that are both costly and time intensive. Discrepancies in access to care due to health inequities such as socioeconomic status or geographic location may lead to vastly different health outcomes. As research informs advances in care, is important to consider inclusion and diversity in JIA research.

**Methods:**

We reviewed and synthesized randomized controlled trials for juvenile idiopathic arthritis, the most common type of arthritis among children and adolescents, in Canada with the aim of characterizing participants and identifying how determinants of health inequities are reported. To do so, we searched Medline (1990 to July 2022), Embase (1990 to July 2022), and CENTRAL (inception to July 2022) for articles meeting all of the following criteria: Canadian randomized controlled trials evaluating pharmacological or non-pharmacological interventions on juvenile idiopathic arthritis populations. Data extraction was guided by the Campbell and Cochrane Equity Methods Group’s PROGRESS-Plus framework on determinants that lead to health inequities (e.g., Place of residence; Race; Occupation; Gender/Sex; Religion; Education; Socioeconomic status; and Social capital).

**Results:**

Of 4,074 unique records, 5 were deemed eligible for inclusion. From these determinants of health inequities, Gender/Sex and Age were the only that were reported in all studies with most participants being female and 12.6 years old on average. In addition, Race, Socioeconomic status, Education and Features of relationships were each reported once in three different studies. Lastly, Place of residence, Occupation, Religion, Social Capital and Time-dependent relationships were not reported at all.

**Conclusions:**

This scoping review suggests limited reporting on determinants of health inequities in randomized controlled trials for JIA in Canada and a need for a reporting framework that reflects typical characteristics of juvenile patient populations.

**Supplementary Information:**

The online version contains supplementary material available at 10.1186/s12969-023-00917-5.

## Introduction

With a disease prevalence of 3 in 1000 Canadian children, juvenile idiopathic arthritis (JIA) is the most common type of arthritis among children and adolescents and is a great cause of pain and discomfort [1]. Studies in the US have shown that within the first year of diagnosis, being non-White and having lower household income are characteristics associated with higher disease activity in children with JIA as well as a longer “time to first appointment” [2, 3]. Furthermore, previous studies have also shown that there is a notable economic burden of having a child with JIA due to costly medications and specialist treatments such as physiotherapy [4, 5]. This is burdensome as specialist treatments are typically not covered by the Canadian healthcare system and there is a lack of consistency in medication coverage across Canadian provinces as it is dependent on the degree of provincial and private insurance coverage.

There are recent calls for consideration of health inequities, that is, differences in health status or differences in the distribution of health resources between groups in society caused by dissimilarity in the social conditions they live [6], in rheumatology research. In a 2019 systematic review of US-based randomized controlled trials (RCTs) in rheumatoid arthritis (RA), Strait et al. characterized participants as mostly females and noted underrepresentation of minority racial and ethnic groups, males, and younger and older individuals [7]. In 2022, we conducted a scoping review of Canadian RCTs in RA patients and similarly found that participants were largely middle-aged, female, and White. In addition, we assessed reporting of determinants of health inequities and found that most frequently reported were sex and age, and to a lesser extent race, education, and socioeconomic status (SES) [8]. To our knowledge, there are no syntheses on how health inequities have been considered in research among patients with JIA. As such, we conducted a scoping review of Canadian RCTs of interventions among JIA patients to characterize participants and assess how determinants of health inequities are reported. We used the Campbell and Cochrane Equity Methods Group’s PROGRESS-Plus Framework which represents factors (“PROGRSS factors”) that lead to inequities in health (i.e., (i.e., **P**lace of residence; **P**lace, **C**ulture, **E**thnicity, **L**anguage; **O**ccupation; **G**ender/sex; **R**eligion; **E**ducation; **S**ocioeconomic status; and **S**ocial capital) Plus additional factors (i.e., Personal characteristics associated with discrimination; Features of relationships; and Time-dependent relationships) [9].

## Methods

### Inclusion criteria

To conduct this scoping review, we followed the Arksey and O’Malley framework [10]. We included studies that: (1) used an RCT design; (2) evaluated interventions, defined as either pharmacological or non-pharmacological treatments or services; (3) included participants with JIA; (4) was conducted in Canada; (5) published in English; and (6) published between 1990 and July 2022. This time period was selected based on our prior scoping review on the reporting of determinants of health inequities in RCTs for RA [8] for consistency and to facilitate comparison and contextualization of findings. Studies were determined to be conducted in Canada if they met the following criteria: (1) Canadian affiliation(s) of lead and senior authors; (2) location(s) of data collection were at Canadian facilities; and (3) lead funders were Canadian-based organizations. We excluded conference abstracts, protocols, and studies with unpublished results. Inclusion was restricted to RCTs as they are recognized as a “gold standard” study design [11] and widely available resources for standardization and reporting facilitate meaningful intertrial comparisons [12].

### Information sources and search

We conducted a search in MEDLINE (Ovid 1990 to July 2022), Embase (Ovid 1990 to July 2022), and CENTRAL (inception-July 2022). The search strategy was adapted from the sensitivity-maximizing Cochrane Highly Sensitive Search Strategy for identifying randomized trials in MEDLINE (2008 revision), and JIA filters based on RA filters used previously [8, 13, 14].

### Selection of studies

All retrieved publications were screened for eligibility, first by title and abstract, then by full text (by NG and MT). Publications deemed eligible for inclusion proceeded to full-text review. Discussion between authors occurred to resolve uncertainty and to achieve consensus about inclusions.

### Data extraction and synthesis of results

We extracted the following data from included studies: authors, title, journal, year, the objective of the study, and study intervention. Of particular interest was the reporting of 11 factors under PROGRESS-Plus: (1) Place of residence; (2) Race, culture, ethnicity, language; (3) Occupation; (4) Gender, sex; (5) Religion; (6) Education; (7) Socioeconomic status; (8) Social capital; (9) Personal characteristics associated with discrimination; (10) Features of relationships; and 11) Time-dependent relationships) [9]. For studies that reported PROGRESS-Plus factors, we extracted further details, such as how these determinants of health inequities were defined and operationalized, and the distribution of study participants according to these determinants.

As this was a scoping review of published literature, there was no participant recruitment and ethics approval was not required.

## Results

### Included studies

**Fig. 1:**
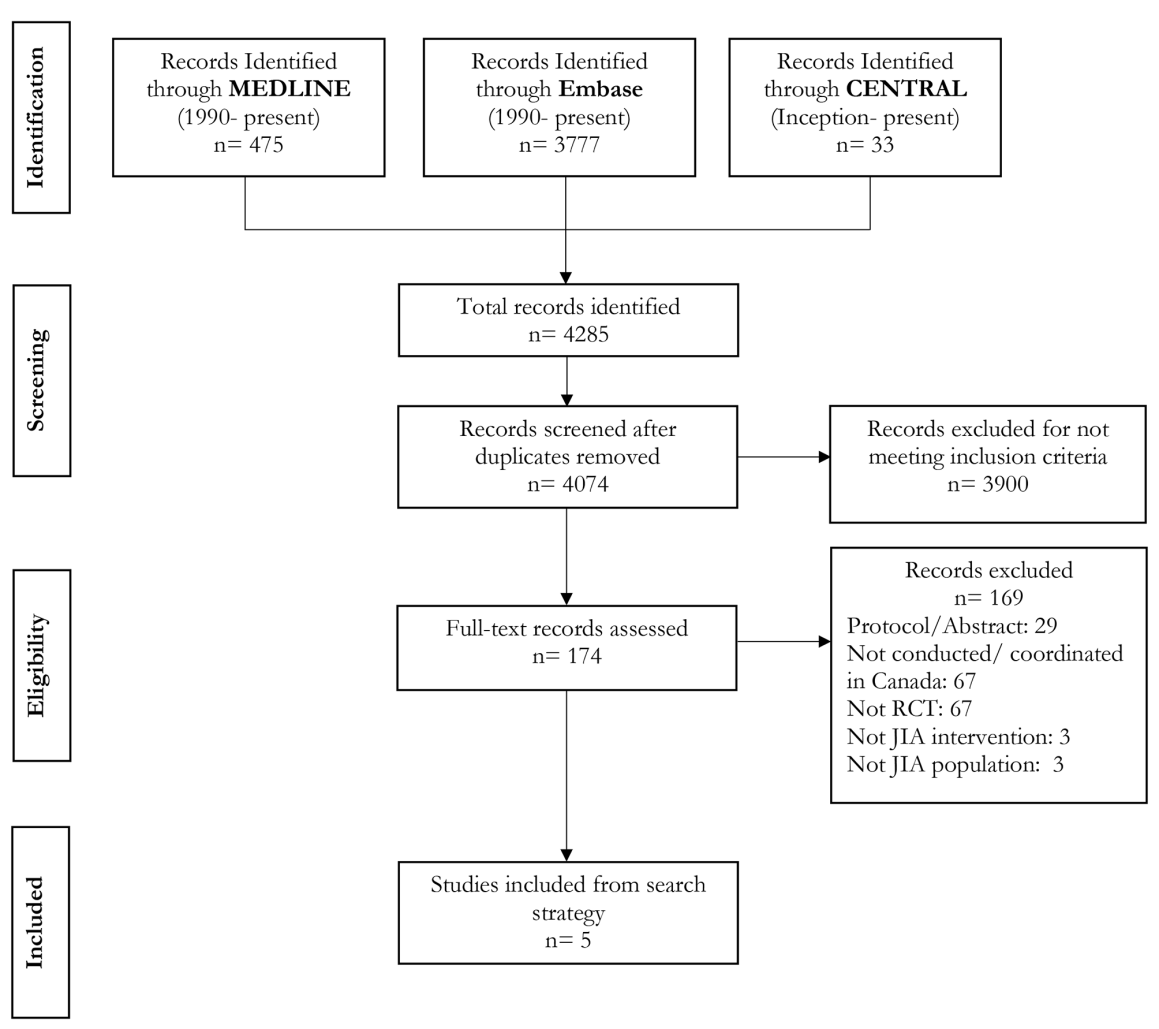
Preferred Reporting Items for Systematic Reviews and Meta-Analyses (PRISMA) diagram. JIA = Juvenile Idiopathic Arthritis; RCT = randomized controlled trial

Our search strategy identified 4,285 records (Fig. 1). Of those records 4,074 were deemed unique and were first reviewed by title and abstract to screen for inclusion criteria, such as being a Canadian RCT and participants having JIA. Studies that passed the initial screening were then reviewed by full text to ensure all studies adhered to the inclusion criteria. At this stage, emphasis was put on determining if Canada was the central site for the JIA RCTs, as many studies had Canadian locations, but few were centrally coordinated by Canadian researchers. Altogether, five studies were deemed eligible, and they ranged in sample size from 14 to 219 participants. From the eligible studies, one evaluated a pharmacological intervention, determining the impact of food on the bioavailability of oral methotrexate (n = 14) [15]. The other four studies evaluated non-pharmacological interventions, namely a smartphone-based pain management program (n = 60) [16], an online peer mentoring program (n = 30) [17], a web-based coping and stress management program (n = 219) [18], and the effectiveness of high-intensity aerobic training compared to low-intensity training on children with JIA (n = 80) [19] (Table 1).

**Table 1:** Characteristics of included studies and reporting of PROGRESS-Plus factors

Study ID	Type of intervention	Intervention	N	Age	%Female	P	R	O	G	R	E	S	S	P1	P2	P3
Dupuis 1995 (13)	Pharmacological	Usual Methotrexate dose was given either orally (fasting and non-fasting) or via IV injection in the morning for 3 consecutive weeks	14	8.39	71.4				X					X		
Singh-Grewal 2007 (17)	Non-pharmacological	High intensity aerobics exercise vs. quigong for 12 weeks, 3 times per week	80	11.6	79.9				X					X		
Stinson 2016 (15)	Non-pharmacological	Video calls with peer mentors (adolescents with managed Juvenile Idiopathic Arthritis )	30	14.1	94.0				X		X			X		
Stinson 2020 (16)	Non-pharmacological	A website containing specific coping and stress management methods compared to a general Juvenile Idiopathic Arthritis education website	219	14.4	70.3				X			X		X	X	
Lalloo 2021 (14)	Non-pharmacological	iCanCope app and app with only symptom tracking	60	15.0	78.3		X		X					X		

X denotes factor reported in the study and blank cells denote factor not reported;

PROGRESS-Plus Factors: **P** (Place of residence), **R** (Race/ethnicity/culture/language), **O** (Occupation), **G** (Gender/sex), **R** (Religion), **E** (Education), **S** (Socioeconomic status), **S** (Social Capital), **P1** (Personal characteristics associated with discrimination), **P2** (Features of relationships), **P3** (Time-dependent relationships).

### Characteristics of participants

We characterized participants of RCTs of JIA in Canada from studies that reported sex (all studies) and age (all studies). Participants were primarily female, comprising 70.3% [18] to 94% [17] of study populations. With respect to age, participants were mainly early adolescents. The average age of participants was 12.6 years, with a range of 8.39 to 15.0 years. Two studies included participants under 12 years old [15, 19], with the youngest being 2.8 years [15]. In the one study that reported on race, 70.2% of participants were White [16].

### Reporting of PROGRESS-Plus factors

We also assessed how included studies reported PROGRESS-Plus factors (summarized in Table 1). Five of the factors - Place of residence, Occupation, Religion, Social Capital and Time-dependent relationships - were not reported in any study. Six factors - Personal Characteristics associated with discrimination, Race, Sex, Socioeconomic status, Education and Features of relationships - were reported but to a varying extent.

Personal characteristics associated with discrimination falls under the “Plus” category of the PROGRESS-Plus factors, and encompass characteristics such as age or disability that can be a cause of discrimination [9]. From the personal characteristics associated with discrimination, the only factor that was reported was age. Though we note that age may not be associated with discrimination in the pediatric demographic. All included studies reported participant ages, four using the mean and standard deviation in years [16–19] and one reporting the age of each participant in years individually [15]. Two studies reported the mean age of participants and controls separately [16, 17], and one study reported the range of ages as well [19].

Within the PROGRESS-Plus factors, the category of race, ethnicity, culture and language refer to the racial, ethnic and cultural background of individuals [9]. This is relevant as it has been historically shown that health outcomes differ when comparing patients across different races, ethnicities, and cultures. The three terms are often used interchangeably, however race refers to a biological quality while ethnicity and culture encompass social aspects [9, 20]. Although the category of race is controversial in nature due to its lack of basis on distinct genetic differences, it is important to note as most racial inequities are caused by the social experiences of “racialized groups” and may impact the inequities seen in healthcare [9]. Race was reported in a single study [16] with a limited diversity of race categories (Aboriginal, Arab or West Asian, Black, Chinese, Filipino, Multiracial, South Asian, South East Asian, and White) and no mention of the related factors of culture and ethnicity. Of note, an inclusion criterion for participation in this study was the ability to speak and read in English however there was no mention of native or additional languages. Most participants were White (70.2%), with the second largest group (10.5% of participants) being Multi-racial. No definition or description was given on what the criteria were to be considered “White” or “Multi-racial, such as place of birth or ancestry.

It is important to consider sex in health research as it results in variation in disease risk and incidence [9]. It is equally important to consider gender as it impacts an individual’s experience of the disease. An understanding of which populations require more personalized care would help improve disease outcomes and allow for better use of resources. As previously described in characterizing trial participants, sex was reported in all studies. When reporting, gender and sex terms were used interchangeably in two studies [15, 19], with both equating “boys” and “girls” (gender) to “male” and “female” (sex). These two studies were conducted between 1995 and 2007. All studies were limited to binary categories (male or female) with no gender or sex-diverse terms (such as gender non-binary or gender fluid).

Socioeconomic status (SES) encompasses income, educational attainment, and occupation (the latter two being independent PROGRESS-Plus factors) [21] and is a measure of an individual’s economic and social status in relation to others [22]. Average household income was reported in one study [18], ranging from <$25,000 CAD to between $100,000 and $150,000 CAD with an option not to report. Few (4.6%) participants fell in the <$25,000 CAD category, 12.7% in the $25,000 to $49,999 CAD, 14.2% in the $50,000 to $74,999 CAD, 16.8% in the $75,000 to $99,999 CAD, 16.8% in the $100,000 to $150,000 CAD, and 22.3% chose not to report. Given the younger age of JIA patients in these trials, an important consideration is who are questions related to income directed to – whether participants themselves or parents/guardians. It is likely that responses regarding household income was reported by parents and/or guardians.

Education is important to consider due to its long-term effects on socioeconomic status (specifically income). An individual’s level of education impacts their level of employment which is generally correlated with income, and those with higher education levels and incomes tend to live healthier lives and experience less financial burden [9]. However, this again, is of particular consideration for younger participants who are still attaining education. Participant education level was only mentioned in one study and was reported as the average current grade of the participants (Grade 9) [17].

Features of relationships refers to the characteristics of the external relationships of a patient that affect their ability to assert their autonomy over their health [23]. These relationships are important to consider as they help to provide context for patient treatment, especially for paediatric patients where parents/guardians hold primary responsibility for patient care. Features of relationships were mentioned in one study as the percentage of caregivers who had graduated from college or graduate school (65.5%) [18] .

## Discussion

Guided by the Campbell and Cochrane Equity Methods Group’s PROGRESS-Plus framework [9], we conducted a scoping review of five RCTs of interventions for individuals with JIA in Canada to improve our understanding of how health inequities are reported. Among PROGRESS-Plus factors, only sex and age were reported in all RCTs. Race and education were reported once by different studies [16, 17]. Socioeconomic status and features of relationships were reported once by the same study [18]. From this limited data, we characterized participants of JIA RCTs in Canada as primarily early-adolescents and female. In showing the very limited reporting of determinants of health inequities in RCTs of JIA in Canada our study has implications for raising awareness of the need of increased representation and diversity in research among this unique patient population.

A key to our scoping review was assessing how PROGRESS-Plus factors were considered in studies that reported them. Although sex was reported in all studies, there were some inconsistencies with the use of sex and gender in some studies [15, 19]. For example, the terms “male” and female” were used when reporting overall patient characteristics however the terms “girl” and “boy” were used when describing participants through the body of the text as equivalent terms, with one study explaining they compared different sexes and used boys and girls as their description. However, conflation of sex and gender terms were not seen in studies published in the 2010s and onwards [16–18]. This is interesting as this pattern is not consistent with reporting trends in studies on RA [8], where more studies published before the year 2000 correctly used the terms sex and gender compared to those after 2000. Age was the other commonly reported PROGRESS-Plus factor, and it was reported as a mean age in years in most studies. JIA is diagnosed following a period of arthritis (joint inflammation) lasting longer than six weeks with an onset age of less than 16 years [24], however depending on the type of JIA (systemic arthritis, oligoarthritis, rheumatic factor (RF) positive arthritis, RF negative arthritis, enthesitis-related arthritis or psoriatic arthritis) the age at onset differs. The age at onset tends to fall under either the ages of 2 to 4 years or between 9 and 12 years, with only RF positive arthritis and enthesitis-related arthritis having an onset during adolescence [25]. The average age of JIA patients is thus well captured in the trials, as the mean age of participants across all studies was 12.6 years, especially as most trial interventions had a focus on patients coping with JIA independently rather than treatment at diagnosis [16–18].

It is also important to assess less frequently reported PROGRESS-Plus factors. Looking at the factor of SES, this was reported in one study as annual household income, according to five categories ranging from <$25,000 CAD to between $100,000 and $150,000 CAD, with an option to not report (22.3% of participants) [18]. The majority of participants who chose to report (77.7%) had average household incomes upwards of $100,000 CAD (the highest income category option). This study was conducted in eight Canadian provinces (Ontario, Alberta, Nova Scotia, Saskatchewan, Quebec, British Colombia, Newfoundland and Labrador, and Manitoba) and patient responses fall in line with epidemiological data found on JIA patients in Manitoba where 65.5% of patients were categorized in the highest income quintiles (Q3-5, no information was given regarding the range of incomes each quintile fell under) [26]. However as average household income is self-reported with an option to decline answering, the reliability of this data is limited as it may be subject to reporting bias. Furthermore, given the age of the JIA patient population, it is likely that parents/guardians are providing this information. Understanding individual patients’ ability to afford treatment is important as it has been shown that there is a substantial cost related to JIA treatment [4, 5]. An accurate awareness of how much of an economic burden JIA care is on families could help pave the way to increased financial support from the healthcare system.

It is difficult to speak on the degree of representation of determinants of health inequity within JIA trials due to the limited published data. The ratio of male to female participants is reflective of the demographic characteristics of JIA, with most trials having females account for about 70% of total participants as the majority of JIA patients tend to be female [26, 27]. Looking at the single study that reported on race [16], there is a significantly higher percentage of White participants (70%) compared to all other races which could indicate a need for increased participant diversity across the board, but without reported data from other trials definitive conclusions cannot be made. Interestingly, no studies reported on the place of residence of participants. Some research has shown that 43% of patients live in rural areas which is particularly relevant at it has been found that individuals in rural Canada tend to have a poorer access to healthcare [28, 29] and JIA is an illness that requires individualized and ongoing treatment. Because of this, it is important to know the place of residence of participants as it may have a direct effect on the quality and effectiveness of interventions due to lack of resources.

Our scoping review has demonstrated the lack of health inequity reporting, as in current conditions, there is insufficient data to determine if RCTs in JIA are being conducted in a manner that is reflective of equity, diversity, and inclusion factors such as race, sex, and socioeconomic status, as well as other PROGRESS-Plus factors. It is important to ensure that RCTs (and other health research studies) are being designed in a manner that would have a benefit to patients from all backgrounds rather than those that are typically represented, such as White and wealthy populations [30, 31]. This raises the question of why there has been such a drastic limit to the number of determinants of inequity being reported in JIA RCTs. It could in part be due to the increased workload of asking what could be considered “invasive” questions such as religion and SES. Factors such as occupation, SES, and time-dependent relationships may seem irrelevant to ask paediatric patients, and it may not be clear that these questions could be asked of the guardians and/or household (for example, asking average household income or marital status of parents/guardians). Due to the impact of parents/guardians as primary caregivers on the management and maintenance of JIA treatment it is important to gain an understanding of the general demographics of these caregivers. Caregiver characteristics were only reported in one study [18] where parent/guardian education level was asked, most likely as caregivers were also asked to participate in the trial. This indicates that clearer guidance should be developed to better encompass the relevant characteristics of paediatric patients and their households.

This scoping review has several limitations. As only RCTs were considered, we may have included a more biased sample of studies that may be considered of higher rigor. This may suggest that our findings underestimate the extent of issues of underreporting of determinants of inequity. However, RCTs may be limited with respect to inclusion criteria, measurement of determinants of inequity (e.g., have comprehensive questionnaires) and time for participants to complete such measures. Future research could consider expanding to expanding to observational studies and point-of-care trials to address determinants of equity. We used the PROGRESS-Plus framework to guide extraction of reporting of determinants of inequities; however, this framework may not be exhaustive, or as previously discussed, entirely aligned with paediatric patient characteristics.

## Conclusions

Ensuring that research in JIA is representative of the entire spectrum of patients is critical as their treatment requires individualized and chronic care. This scoping review allowed us to identify that determinants of inequities are not commonly measured in Canadian RCTs for JIA interventions, which could be problematic when translating study findings to impact care. We were also able to identify that inequity-based health determinant factors should be adjusted to better suit paediatric populations to improve reporting. Overall, considering determinants of inequities is integral to facilitating relevant research needed to improve the outcomes and quality of life for Canadian JIA patients.

### Electronic supplementary material

Below is the link to the electronic supplementary material.

Supplementary Material 1

## Data Availability

All data generated and analyzed during this study are included in the published article (see **Table 1**).

## Abbreviations

JIA: Juvenile Idiopathic Arthritis
RCT: Randomized Controlled Trial
RA: Rheumatoid Arthritis
PROGRESS: Place of residence, Culture, Ethnicity, Language, Occupation, Gender/sex, Religion, Education, Socioeconomic status, Social Capital
CAD: Canadian dollar
RF: Rheumatic factor
