# Supplementary material for: Reporting of determinants of health inequities and participant characteristics in randomized controlled trials of juvenile idiopathic arthritis in Canada: a scoping review
Source: Pediatr Rheumatol Online J. 2023 Nov 6;21:134. doi: 10.1186/s12969-023-00917-5 (PMC10629131; doi:10.1186/s12969-023-00917-5)
Supplement: Supplementary file 1 — Supplementary Material 1 [file 12969_2023_917_MOESM1_ESM.docx]

**Supplementary Materials: Search Strategies**

**Table 2. MEDLINE (Ovid) search strategy**

Ovid MEDLINE(R) and Epub Ahead of Print, In-Process, In-Data-Review & Other Non-Indexed Citations, Daily and Versions(R) <1946 to December 08, 2021>

| **Line number** | **Search terms** | **Number of results** |
| --- | --- | --- |
| 1 | exp arthritis, rheumatoid/ | 118960 |
| 2 | ((rheumatoid or reumatoid or revmatoid or rheumatic or reumatic or revmatic or rheumat$ or reumat$ or revmarthrit$) adj3 (arthrit$ or artrit$ or diseas$ or condition$ or nodule$)).tw. | 139323 |
| 3 | 1 or 2 | 179099 |
| 4 | randomized controlled trial.pt. | 552220 |
| 5 | controlled clinical trial.pt. | 94571 |
| 6 | randomized.ab. | 543058 |
| 7 | placebo.ab. | 223786 |
| 8 | drug therapy.fs. | 2410236 |
| 9 | randomly.ab. | 371448 |
| 10 | trial.ab. | 578858 |
| 11 | groups.ab. | 2282135 |
| 12 | or/4-11 | 5195338 |
| 13 | exp animals/ not humans.sh. | 4923958 |
| 14 | 12 not 13 | 4520296 |
| 15 | 3 and 14 | 56801 |
| 16 | exp Canada/ | 171404 |
| 17 | (canad* or british columbia or alberta or saskatchewan or manitoba or ontario or quebec or new brunswick or prince edward island or nova scotia or newfoundland or labrador or nunavut or northwest territories or yukon).af. | 1287795 |
| 18 | 16 or 17 | 1287795 |
| 19 | 15 and 18 | 4783 |
| 20 | limit 19 to yr="1990 -Current" | 4147 |

**Table 3. Embase (Ovid) search strategy**

Embase <1974 to 2021 December 08>

| **Line number** | **Search terms** | **Number of results** |
| --- | --- | --- |
| 1 | exp arthritis, rheumatoid/ | 216087 |
| 2 | ((rheumatoid or reumatoid or revmatoid or rheumatic or reumatic or revmatic or rheumat$ or reumat$ or revmarthrit$) adj3 (arthrit$ or artrit$ or diseas$ or condition$ or nodule$)).tw. | 206357 |
| 3 | 1 or 2 | 270454 |
| 4 | random$.tw. | 1729445 |
| 5 | factorial$.tw. | 42476 |
| 6 | crossover$.tw. | 82392 |
| 7 | cross over.tw. | 34651 |
| 8 | cross-over.tw. | 34651 |
| 9 | placebo$.tw. | 335054 |
| 10 | (doubl$ adj blind$).tw. | 225558 |
| 11 | (single$ adj blind$).tw. | 27938 |
| 12 | assign$.tw. | 437015 |
| 13 | allocat$.tw. | 174708 |
| 14 | volunteer$.tw. | 274177 |
| 15 | crossover procedure/ | 68813 |
| 16 | double blind procedure/ | 190175 |
| 17 | randomized controlled trial/ | 685837 |
| 18 | single blind procedure/ | 44521 |
| 19 | or/4-18 | 2587125 |
| 20 | 3 and 19 | 25085 |
| 21 | exp Canada/ | 196338 |
| 22 | (canad* or british columbia or alberta or saskatchewan or manitoba or ontario or quebec or new brunswick or prince edward island or nova scotia or newfoundland or labrador or nunavut or northwest territories or yukon).af. | 1826357 |
| 23 | 21 or 22 | 1826357 |
| 24 | 20 and 23 | 3086 |
| 25 | limit 24 to yr="1990 -Current" | 2941 |

**Table 4. Cochrane Central Register of Controlled Trials (CENTRAL) (via Ovid) search strategy**

EBM Reviews - Cochrane Central Register of Controlled Trials <December 8 2021>

| **Line number** | **Search terms** | **Number of results** |
| --- | --- | --- |
| 1 | exp arthritis, rheumatoid/ | 6337 |
| 2 | ((rheumatoid or reumatoid or revmatoid or rheumatic or reumatic or revmatic or rheumat$ or reumat$ or revmarthrit$) adj3 (arthrit$ or artrit$ or diseas$ or condition$ or nodule$)).tw. | 18611 |
| 3 | 1 or 2 | 19470 |
| 4 | exp Canada/ | 3902 |
| 5 | (canad* or british columbia or alberta or saskatchewan or manitoba or ontario or quebec or new brunswick or prince edward island or nova scotia or newfoundland or labrador or nunavut or northwest territories or yukon).af. | 56168 |
| 6 | 4 or 5 | 56168 |
| 7 | 3 and 6 | 759 |
| 8 | limit 7 to yr="1990 -Current" | 734 |
